# Antiviral Prescription in Children With Influenza in US Emergency Departments: New Vaccine Surveillance Network (NVSN), 2016–2020

**DOI:** 10.1111/irv.70124

**Published:** 2025-06-10

**Authors:** Tess Stopczynski, Justin Z. Amarin, James W. Antoon, Olla Hamdan, Laura S. Stewart, James Chappell, Andrew J. Spieker, Eileen J. Klein, Janet A. Englund, Geoffrey A. Weinberg, Peter G. Szilagyi, John V. Williams, Marian G. Michaels, Julie A. Boom, Leila C. Sahni, Mary Allen Staat, Elizabeth P. Schlaudecker, Jennifer E. Schuster, Rangaraj Selvarangan, Christopher J. Harrison, Heidi L. Moline, Ariana P. Toepfer, Angela P. Campbell, Samantha M. Olson, Natasha B. Halasa

**Affiliations:** ^1^ Department of Biostatistics Vanderbilt University Medical Center Nashville Tennessee USA; ^2^ Department of Pediatrics Vanderbilt University Medical Center Nashville Tennessee USA; ^3^ Seattle Children's Research Institute Seattle Washington USA; ^4^ University of Rochester School of Medicine and Dentistry & UR‐Golisano Children's Hospital Rochester New York USA; ^5^ University of California at Los Angeles (UCLA) Los Angeles CA USA; ^6^ Department of Pediatrics University of Wisconsin School of Medicine and Public Health Madison WI USA; ^7^ UPMC Children's Hospital of Pittsburgh, University of Pittsburgh School of Medicine Pittsburgh Pennsylvania USA; ^8^ Texas Children's Hospital and Baylor College of Medicine Houston Texas USA; ^9^ Cincinnati Children's Hospital Medical Center, University of Cincinnati, College of Medicine Cincinnati Ohio USA; ^10^ Children's Mercy Hospital Kansas City Missouri USA; ^11^ Centers for Disease Control and Prevention Atlanta Georgia USA

**Keywords:** antivirals, influenza, pediatrics

## Abstract

**Background:**

Influenza contributes to a high burden of pediatric emergency department (ED) visits annually. Guidelines recommend outpatient antiviral treatment for children at higher risk of severe influenza and recommend considering treatment for those who present within 2 days of symptom onset. We describe antiviral prescription in children with influenza presenting to the ED.

**Methods:**

We analyzed data from the New Vaccine Surveillance Network (2016–2020), including children presenting to the ED and enrolled with confirmed influenza at one of seven pediatric academic centers. We compared characteristics of children prescribed antivirals to those who were not, using generalized estimating equations models to identify predictors of antiviral prescription. Children were considered at higher risk of severe influenza if they were < 5 years old or had an underlying condition.

**Results:**

Overall, 2472 (15%) of 16,915 enrolled children tested positive for influenza virus. Among these, 1931 (78%) were at higher risk of severe influenza; only 622 (32%) received an antiviral. Among 233 (9%) children not at high risk with symptom onset ≤ 2 days, 62 (27%) were prescribed an antiviral. Children prescribed an antiviral had a shorter duration of illness prior to presenting to the ED. For children at higher risk of severe influenza, odds of antiviral prescription were higher for those clinically tested for influenza and with underlying conditions.

**Conclusion:**

Clinical testing and having an underlying condition were associated with antiviral prescription in children at higher risk of severe influenza. However, only 1/3 of those at higher risk were prescribed an antiviral. Strategies to increase antiviral use for children at higher risk for influenza in the ED are needed.

## 1 Introduction

Influenza causes substantial morbidity and mortality, particularly in vulnerable populations such as young children and those with certain underlying medical conditions [1]. The Centers for Disease Control and Prevention (CDC) estimated that between 2016 and 2020, influenza accounted for approximately 162,535 hospitalizations and roughly 1679 deaths among children < 18 years of age [2]. Pediatric influenza‐related disease can range from mild respiratory symptoms to severe outcomes such as pneumonia, encephalitis, and death [3]. Prompt identification and treatment of children with influenza is crucial in reducing illness severity.

Influenza‐specific antiviral medications have been shown to reduce the duration and severity of influenza symptoms when used early in the course of illness, as well as reduce the risk of severe illness and reduce intensive care unit (ICU) stays [4, 5]. The American Academy of Pediatrics (AAP), Infectious Diseases Society of America (IDSA), and CDC recommend treating suspected or confirmed influenza in non‐hospitalized children who are at higher risk of influenza complications; namely, children < 5 years old, especially those < 2 years, and those with certain underlying medical conditions. For patients not at higher risk of complications, clinicians may consider antiviral treatment for outpatients with suspected influenza who present within 2 days of symptom onset and children with household contacts who are at higher risk for severe influenza, such as pregnant women [6].

Outpatient settings, including the emergency department (ED), provide an ideal opportunity for early antiviral treatment because they are a frequent location of care for children with influenza‐like illness. Few studies document the frequency of antiviral prescriptions for children with influenza or describe the characteristics of those receiving antivirals at the time of an ED visit. Some studies suggest that antiviral prescription is infrequent in outpatient settings, even when children present within 2 days of symptom onset [7, 8]. We analyzed data from the CDC‐funded New Vaccine Surveillance Network (NVSN) that performs systematic influenza research testing for children presenting with fever or an acute respiratory illness (ARI) symptom. The aim of this study was to evaluate antiviral prescription in children presenting to the ED with laboratory‐confirmed influenza over four influenza seasons.

## 2 Methods

### 2.1 Study Population

Between December 1, 2016, and March 31, 2020, children < 18 years old who were evaluated at the ED at one of seven NVSN ARI surveillance sites (Vanderbilt University Medical Center [VUMC; Nashville, Tennessee]; Golisano Children's Hospital [Rochester, New York]; Cincinnati Children's Hospital Medical Center [Cincinnati, Ohio]; Texas Children's Hospital [Houston, Texas]; Seattle Children's Hospital [Seattle, Washington]; Children's Mercy Hospital [Kansas City, Missouri]; and UPMC Children's Hospital of Pittsburgh [Pittsburgh, Pennsylvania]) were approached for enrollment if they met eligibility criteria. Between December 2016 and November 2019, three sites restricted enrollment in the ED to children less than 5 years of age from December to June of each year; the other study months include all children < 18 years. Eligible children resided in the catchment area of the respective site and met the previously described NVSN inclusion criteria [9]. For this analysis, we further excluded enrolled children if they were admitted to the hospital or if their treatment status included influenza antivirals prior to the enrollment visit or was unknown (*n* = 172). The institutional review boards at each of the seven sites and CDC approved the study protocol.

### 2.2 Data and Specimen Collection

The research teams at each site obtained written informed consent from parents or guardians, and assent from children, if applicable, and administered a standardized questionnaire to collect sociodemographic and clinical information. Medical chart reviews were conducted to record physical examination findings, underlying medical conditions, and measures of disease severity. Documentation was recorded regarding whether patients received antivirals during the visit (oseltamivir, zanamivir, baloxavir, and peramivir), either by administration or prescription at discharge. History of previous influenza vaccination was also documented and verified in the vaccine registry. Symptom duration was determined by calculating the days between symptom onset date (collected in the interview) and the date of the interview.

Mid‐turbinate nasal and/or oropharyngeal research specimens were collected from patients. Specimens underwent commercial or institution‐specific in‐house reverse transcription‐polymerase chain reaction assays (RT‐PCR) at each study site for influenza virus type and subtype [9]. In cases where study specimens could not be collected, clinically ordered respiratory specimens from provider‐ordered testing were salvaged within 2 days and tested with the same platforms as described above. Information about additional clinical virus testing conducted for routine medical care was gathered from participants' electronic medical records. This clinical testing included: 1) rapid antigen influenza diagnostic testing (RIDT), 2) rapid or conventional nucleic acid amplification tests (NAATs), and 3) NAATs panel testing. Our main analysis used clinical and research RT‐PCR testing results to classify influenza‐positive patients.

### 2.3 Children at Higher Risk for Severe Influenza

Children were defined as at higher risk for severe influenza based on AAP, IDSA, and CDC guidance and included those who were < 5 years old or had one of the following underlying medical conditions: respiratory disease, cardiovascular disease, kidney disease, hepatic disease, hematologic disease, metabolic disorder, neurologic disorder, or immunosuppression [6]. Other conditions that indicate higher risk for severe influenza, such as pregnancy, were not included in this analysis due to the low frequency of these conditions in children or lack of assessment in this study. Antiviral prescription among children who did not meet the definition of high risk for severe influenza but had symptom onset within 2 days of illness was also assessed separately.

### 2.4 Statistical Methods

Demographic and clinical characteristics (age, sex, race/Hispanic origin, study site, insurance status, clinical influenza testing, symptoms at presentation, underlying medical conditions, influenza season, and peak of influenza season) were summarized as absolute and relative frequencies for categorical variables and median (interquartile range, IQR) for continuous variables. These characteristics were compared between influenza‐positive children prescribed antivirals and influenza‐positive children without antiviral prescription using logistic regression for binary variables, multinomial logistic regression for multinomial variables, and linear regression for continuous variables. All analyses including robust standard errors accounting for clustering by patient identifier. Additionally, we assessed antiviral prescription status stratified by higher risk of severe influenza illness and by symptom onset within 2 days. We defined influenza season (e.g. 2016–2017) as the 12‐month period from the first Sunday in July for a given year to the day before the first Sunday in July of the following calendar year. Influenza season peak was defined for each site as the 13 consecutive weeks that contained the maximum cumulative number of influenza cases [10].

We used a generalized estimating equations model with a logistic link function to evaluate factors associated with odds of antiviral prescription among children with influenza. This method accounts for the correlated nature of the data using the individual level identifier (2% of observations in the analytic dataset came from patients who had more than one unique encounter over the four included influenza seasons). Our model was restricted to children at higher risk of severe illness and included the following variables: age (years), vomiting or diarrheal symptoms (yes or no), onset of symptoms ≤ 2 days (yes or no), influenza tested clinically (yes or no), ≥ 1 underlying medical condition as previously defined (yes or no), influenza season (2016–2017, 2017–2018, 2018–2019, and 2019–2020), influenza season peak (yes or no), receipt of current season influenza vaccine (yes or no), and study site, with individual level identifiers to account for repeat encounters. These variables were chosen a priori based on study authors' opinion regarding reasons for prescription. We used multiple imputation by chained equations with M = 20 iterations to address missing data (*n* = 36). Adjusted odds ratios and their associated 95% confidence interval were estimated for each predictor. Statistical significance was based on a nominal level of α = 0.05 (two‐tailed, if applicable). All analyses were performed using R version 4.3.0.

## 3 Results

### 3.1 Study Population

**FIGURE 1:**
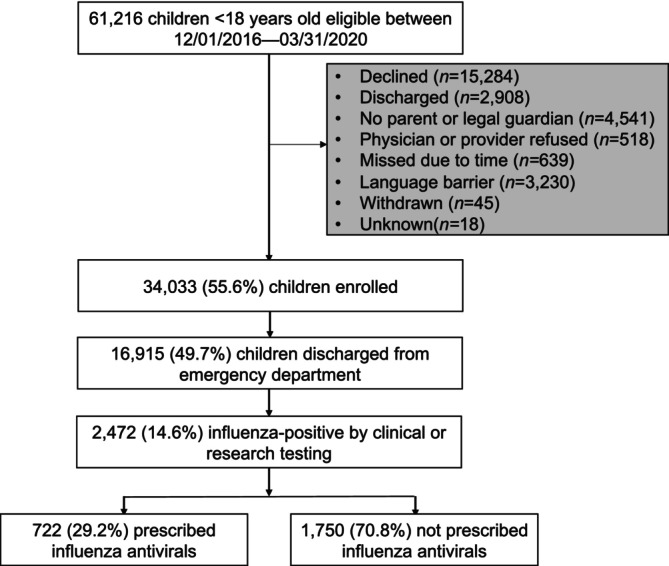
Flow diagram of study participants between 12/01/2016 and 03/31/2020.

Of 16,915 pediatric ED participants who met inclusion criteria and were enrolled, 2472 (14.6%) tested positive for influenza virus by clinical or research molecular testing (Figure 1), with 53.0% receiving clinical influenza testing. ED participants had a median age of 3.6 years; 54.2% were male and 51.0% non‐Hispanic Black (Table 1). Overall, 722 (29.2%) children were prescribed an antiviral, of which oseltamivir was the only antiviral prescribed (Figure 1). Furthermore, among the 2107 cases that occurred in the influenza season peak, antivirals were prescribed more frequently during the mid‐to‐late peak season. Prescribing was also higher overall during the later influenza seasons, with the 2019–2020 influenza season having the highest proportion of prescriptions (Table 1).

**TABLE 1:** Demographics and clinical characteristics of children with influenza by clinical or research testing in the emergency department, stratified by prescription of influenza antivirals.

Characteristic	Overall, *N* = 2472	Antiviral prescription, *n* = 722	No antiviral prescription, *n* = 1750	*p* value^a^
Age at screening (years)—median (IQR)	3.6 (1.7, 6.4)	2.9 (1.3, 5.9)	3.9 (1.9, 6.6)	**< 0.001**
Age group at screening—*n* (%)				
0 to < 1 year old	334 (13.5)	141 (19.5)	193 (11.0)	
1 to < 2 years old	396 (16.0)	126 (17.5)	270 (15.4)	
2 to < 5 years old	908 (36.7)	238 (33.0)	670 (38.3)	
≥ 5 years old	834 (33.7)	217 (30.1)	617 (35.3)	
Male—*n* (%)	1340 (54.2)	382 (52.9)	958 (54.7)	0.41
Race and Hispanic origin—*n* (%)				0.05
Hispanic	676/2441 (27.7)	223/709 (31.5)	453/1732 (26.2)	
Non‐Hispanic White	339/2441 (13.9)	97/709 (13.7)	242/1732 (14.0)	
Non‐Hispanic Black	1245/2441 (51.0)	336/709 (47.4)	909/1732 (52.5)	
Non‐Hispanic other	181/2441 (7.4)	53/709 (7.5)	128/1732 (7.4)	
Study site—*n* (%)				**< 0.001**
A	789 (31.9)	302 (41.8)	487 (27.8)	
B	258 (10.4)	9 (1.2)	249 (14.2)	
C	397 (16.1)	95 (13.2)	302 (17.3)	
D	185 (7.5)	95 (13.2)	90 (5.1)	
E	163 (6.6)	65 (9.0)	98 (5.6)	
F	432 (17.5)	119 (16.5)	313 (17.9)	
G	248 (10.0)	37 (5.1)	211 (12.1)	
Insurance status—*n* (%)				0.45
Private	255/2438 (10.5)	77/709 (10.9)	178/1729 (10.3)	
Public	1984/2438 (81.4)	565/709 (79.7)	1419/1729 (82.1)	
Public and private	13/2438 (0.5)	5/709 (0.7)	8/1729 (0.5)	
Self‐pay	186/2438 (7.6)	62/709 (8.7)	124/1729 (7.2)	
Clinical testing for influenza—*n* (%)	1310 (53.0)	653 (90.4)	657 (37.5)	**< 0.001**
Influenza positive by clinical testing	1176/1310 (89.8)	642/653 (98.3)	534/657 (81.3)	**< 0.001**
Duration since symptom onset—median (IQR)	2.0 (1.0, 3.0)	1.0 (1.0, 2.0)	2.0 (1.0, 4.0)	**< 0.001**
Diarrhea—*n* (%)	457/2465 (18.5)	115/717 (16.0)	342/1748 (19.6)	**0.04**
Vomiting—*n* (%)	512/2462 (20.8)	150/718 (20.9)	362/1744 (20.8)	0.96
Fever—*n* (%)	2330/2456 (94.9)	694/715 (97.1)	1636/1741 (94.0)	**0.01**
Influenza season—*n* (%)				**< 0.001**
2016–2017	526 (21.3)	70 (9.7)	456 (26.1)	
2017–2018	633 (25.6)	183 (25.3)	450 (25.7)	
2018–2019	543 (22.0)	191 (26.5)	352 (20.1)	
2019–2020	770 (31.1)	278 (38.5)	492 (28.1)	
Influenza season peak^b^—*n* (%)	2107 (85.2)	636 (88.1)	1471 (84.1)	**0.01**
Early peak	590/2107 (28.0)	167/636 (26.3)	423/1471 (28.8)	
Mid peak	875/2107 (41.5)	266/636 (41.8)	609/1471 (41.4)	
Late peak	642/2107 (30.5)	203/636 (31.9)	439/1471 (29.8)	
Receipt of current influenza vaccine—*n* (%)	964/2440 (39.5)	310/711 (43.6)	654/1729 (37.8)	**0.01**

^a^ *p* Values were calculated using linear regression for continuous outcomes, logistic regression for binary outcomes, and multinomial logistic regression for multinomial outcomes; all included robust standard errors adjusted for clustering by patient identifier.

^b^ Influenza season peak was defined for each site as the 13 consecutive weeks that contained the maximum cumulative number of influenza cases.

### 3.2 Antiviral Prescription Among Children at Higher Risk for Severe Influenza Illness

Among the 2472 children with laboratory‐confirmed influenza, 1931 (78.1%) were classified as being at higher risk for developing severe illness. Among children at higher risk of severe influenza illness, 622 (32.2%) were prescribed an antiviral. Among children < 6 months old who are at higher risk of influenza and who are ineligible for influenza vaccination, 51% were prescribed antivirals. In unadjusted analyses, those prescribed antivirals were more frequently clinically tested for influenza and more likely to have positive results by clinical testing compared to children who were not prescribed an antiviral (*p* < 0.001, Table 2). Furthermore, children prescribed antivirals had a shorter median duration between symptom onset and presentation, more frequently received current season influenza vaccine, and more frequently had an underlying cardiovascular or oncologic/immune condition compared to those who were not prescribed an antiviral (*p* < 0.001, *p* = 0.03, *p* = 0.001, and *p* < 0.001, respectively). The percentage of children at higher risk who received antiviral prescriptions was highest during the 2019–2020 influenza season compared to previous seasons.

**TABLE 2:** Demographic and clinical characteristics of children with influenza by clinical or research testing, stratified by risk of severe influenza illness and antiviral prescription.

Characteristic	Children at higher risk of severe influenza illness^a^			Children not at higher risk with symptom onset ≤ 2 days^b^		
	Antiviral prescription, *n* = 622	No antiviral prescription, *n* = 1309	*p* value^c^	Antiviral prescription, *n* = 62	No antiviral prescription, *n* = 171	*p* value^c^
Age at screening (years)—median (IQR)	2.4 (1.1, 4.5)	2.9 (1.4, 4.3)	0.94	7.5 (6.0, 10.8)	8.0 (6.4, 10.9)	0.29
Age group at screening—*n* (%)						
0 to < 1 year old	141 (22.7)	193 (14.7)		—	—	
1 to < 2 years old	126 (20.3)	270 (20.6)		—	—	
2 to < 5 years old	238 (38.3)	670 (51.2)		—	—	
≥ 5 years old	117 (18.8)	176 (13.4)		62 (100.0)	171 (100.0)	
Male—*n* (%)	335 (53.9)	732 (55.9)	0.39	29 (46.8)	88 (51.5)	0.53
Race and Hispanic origin—*n* (%)			0.10			0.10
Hispanic	181/611 (29.6)	316/1296 (24.4)		27/60 (45.0)	49/169 (29.0)	
Non‐Hispanic White	82/611 (13.4)	181/1296 (14.0)		9/60 (15.0)	22/169 (13.0)	
Non‐Hispanic Black	300/611 (49.1)	698/1296 (53.9)		21/60 (35.0)	88/169 (52.1)	
Non‐Hispanic other	48/611 (7.9)	101/1296 (7.8)		3/60 (5.0)	10/169 (5.9)	
Study site—*n* (%)			**< 0.001**			—
A	242 (38.9)	280 (21.4)		37 (59.7)	85 (49.7)	
B	7 (1.1)	177 (13.5)		0 (0.0)	25 (14.6)	
C	81 (13.0)	216 (16.5)		7 (11.3)	41 (24.0)	
D	95 (15.3)	88 (6.7)		0 (0.0)	1 (0.6)	
E	52 (8.4)	72 (5.5)		9 (14.5)	5 (2.9)	
F	108 (17.4)	277 (21.2)		9 (14.5)	13 (7.6)	
G	37 (5.9)	199 (15.2)		0 (0.0)	1 (0.6)	
Insurance status—*n* (%)			0.60			—
Private	72/612 (11.8)	150/1293 (11.6)		3 (4.8)	8/169 (4.7)	
Public	488/612 (79.7)	1053/1296 (81.4)		51 (82.3)	151/169 (89.3)	
Public and private	5/612 (0.8)	6/1293 (0.5)		0 (0.0)	0 (0.0)	
Self‐pay	47/612 (7.7)	84/1296 (6.5)		8 (12.9)	10/169 (5.9)	
Clinical testing for influenza—*n* (%)	566 (91.0)	449 (34.3)	**< 0.001**	54 (87.1)	91 (53.2)	**< 0.001**
Influenza positive by clinical testing	559/566 (98.8)	363/449 (80.8)	**< 0.001**	50/54 (92.6)	73/91 (80.2)	**< 0.001**
Duration since symptom onset—median days (IQR)	2.0 (1.0, 3.0)	3.0 (2.0, 4.0)	**< 0.001**	2.0 (1.0, 2.0)	2.0 (1.0, 2.0)	0.70
Diarrhea—*n* (%)	111/618 (18.0)	282/1308 (21.6)	0.06	2 (3.3)	18 (10.5)	0.10
Vomiting—*n* (%)	134/618 (21.7)	269/1305 (20.6)	0.62	12 (19.4)	37 (21.6)	0.71
Fever—*n* (%)	598/616 (97.1)	1232/1304 (94.5)	0.06	59/61 (96.7)	160/168 (95.2)	0.65
Underlying medical condition—*n* (%)	275 (44.2)	521 (39.8)	0.07	8 (12.9)	29 (17.0)	0.46
Respiratory	146 (23.5)	261 (19.9)	0.07	—	—	
Cardiovascular	26 (4.2)	22 (1.7)	**0.001**	—	—	
Neurologic/neuromuscular	20 (3.2)	27 (2.1)	0.13	—	—	
Hematologic	22 (3.5)	30 (2.3)	0.12	—	—	
Oncologic/immune	15 (2.4)	4 (0.3)	**< 0.001**	—	—	
Gastrointestinal/hepatic	17 (2.7)	32 (2.4)	0.71	—	—	
Genetic/metabolic	24 (3.9)	52 (4.0)	0.90	—	—	
Endocrine	3 (0.5)	12 (0.9)	0.32	—	—	
Renal/urologic	4 (0.6)	8 (0.6)	0.93	—	—	
Influenza season—*n* (%)			**< 0.001**	—	—	**0.003**
2016–2017	60 (9.6)	354 (27.0)		4 (6.5)	51 (29.8)	
2017–2018	161 (25.9)	350 (26.7)		14 (22.6)	38 (22.2)	
2018–2019	164 (26.4)	272 (20.8)		18 (29.0)	24 (14.0)	
2019–2020	237 (38.1)	333 (25.4)		26 (41.9)	58 (33.9)	
Influenza season peak^d^—*n* (%)	550 (88.4)	1116 (85.3)	0.06	55 (88.7)	142 (83.0)	0.29
Receipt of current influenza vaccine—*n* (%)	279/612 (45.6)	520/1292 (40.2)	**0.03**	21/61 (34.4)	50/170 (29.4)	0.50

^a^ Children at higher risk of severe influenza illness are those < 5 years old or with certain underlying medical condition(s).

^b^ Children with symptom onset ≤ 2 days refers to the group of children who were not at increased risk of severe influenza illness but presented to the ED within 2 days of symptom onset (all children ≥ 5 and < 18 years old).

^c^ *p* Values were calculated using linear regression for continuous outcomes, logistic regression for binary outcomes, and multinomial logistic regression for multinomial outcomes; all included robust standard errors adjusted for clustering by patient identifier. Cells marked “—” indicate *p* value cannot be computed due to zero cell counts.

^d^ Influenza season peak was defined for each site as the 13 consecutive weeks that contained the maximum cumulative number of influenza cases.

**FIGURE 2:**
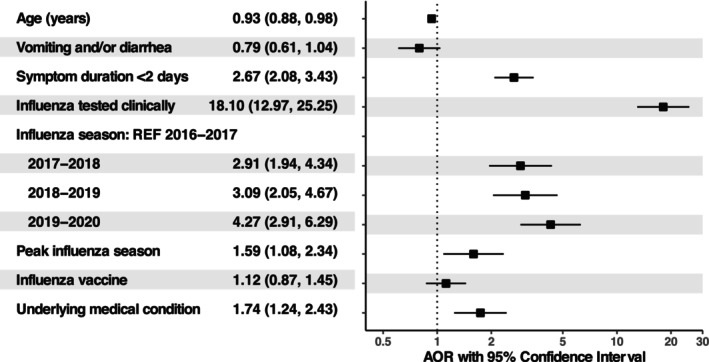
Adjusted odds ratios of antiviral prescription among children at higher risk of severe influenza illness presenting to the emergency departments of seven children's hospitals within the New Vaccine Surveillance Network (*N* = 1931). We used a generalized estimating equations model with a logistic link to compare the odds of antiviral prescription for each of the following variables adjusting for all other variables in the model: age, gastrointestinal symptoms, symptom duration, clinical influenza testing (rapid antigen or PCR) before antiviral prescription, influenza season, influenza season peak, underlying medical condition, receipt of current season influenza vaccine, study site, and individual level identifier.

In the multivariable analysis, younger age, symptom duration within 2 days of enrollment, clinical influenza testing, influenza season, influenza season peak, and having an underlying medical condition were associated with antiviral prescription among children at higher risk of severe influenza in the ED (Figure 2). Older age was associated with lower odds of antiviral prescription (aOR = 0.93, 95% CI = 0.88, 0.98, Figure 2). Significant differences in antiviral prescriptions were observed across study sites, with the likelihood of receiving antivirals ranging from about 8 to 59 times higher compared to the reference site (Table S1). Further, children who underwent clinical testing for influenza had significantly higher odds of being prescribed antivirals compared to those who did not undergo clinical testing (aOR = 18.10, 95% CI = 12.97, 25.25). In addition, the three consecutive influenza seasons from 2017 to 2020 all had higher odds of prescription compared to the 2016–2017 reference season.

### 3.3 Antiviral Prescription Among Children Not at Higher Risk

Among 233 (9.4%) children not classified as higher risk of severe influenza but presenting within 2 days of symptom onset, 62 (26.6%) were prescribed antivirals. Similar to the group at higher risk, the unadjusted analyses showed that children who were prescribed antivirals were more often clinically tested for influenza and were more likely to test positive clinically compared to children who were not prescribed antivirals (Table 2). Temporal treatment differences are also present in this group, with the highest frequency of antivirals prescribed in the 2019–2020 season (41.9%) and the lowest in 2016–2017 season (6.5%) (Table 2).

## 4 Discussion

Using data from the multicenter NVSN platform encompassing children with laboratory‐confirmed influenza over a span of four influenza seasons, our findings provide insight into the recent landscape of antiviral prescription in the ED. Despite treatment guidelines from the CDC and several professional societies advocating for the use of antivirals in children with suspected or confirmed influenza who are at higher risk for severe influenza disease and to consider antivirals for those presenting with symptoms within 2 days of illness onset, we found a substantial gap between recommendations and real‐world treatment practices. Further, only 51% of children < 6 months old who are ineligible for influenza vaccination were prescribed antivirals. The low adherence to guidelines for the treatment of non‐hospitalized children with influenza highlights the need for a better understanding of the factors contributing to these prescribing patterns.

Our data highlight the low overall use of antiviral treatments in children with influenza. Although data are limited, a study conducted across five ambulatory care centers in the United States found that only 15% of children with influenza received an antiviral, while a slightly higher rate of 19% was observed among influenza‐positive children at higher risk of severe influenza illness [11]. Additionally, in a study examining adherence to antiviral treatment guidelines in outpatient children 1–18 years old at higher risk for influenza‐related illness, researchers found that during the 2016–2019 influenza seasons, only 58.1% of the 274,213 children with influenza received antiviral treatment [8]. These observations underscore the need for improved treatment strategies for timely and more comprehensive antiviral treatment for patients at higher risk of severe influenza. Additionally, in a study employing an influenza transmission model, the reduction in hospitalizations doubled when antiviral treatment was administered to individuals at higher risk within 2 days of their symptom onset, providing additional support for use of antivirals to reduce the incidence of severe influenza [12].

We found that children who underwent clinical testing for influenza, likely due to the physician's suspicion of influenza based on symptoms, had 13–25 times higher adjusted odds of being prescribed antivirals as compared to children who were not clinically tested, regardless of higher‐risk status. NVSN has previously identified suboptimal use of clinical influenza testing among hospitalized children during the 2015–2018 influenza seasons and the resultant underdiagnosis of laboratory‐confirmed influenza [13]. Given our findings, we surmise that this problem creates a cascade effect: lack of clinical testing leads to underdiagnosis of influenza cases, while lack of empiric treatment based on symptoms alone results in insufficient antiviral treatment for many suspected cases, despite outpatient recommendations to treat suspected influenza. Our findings align with other studies that have shown an increase in antiviral therapy with the implementation of influenza rapid PCR testing in the ED [14, 15]. One study found that antiviral prescriptions increased from 24.2% to 61.1% with rapid PCR testing [14]. However, only 53.0% of the children in our study received clinical influenza testing. Our findings emphasize the importance of increasing compliance with current recommendations to guide appropriate antiviral treatment in pediatric patients presenting with ARI; testing is not needed to treat with antivirals if there is suspected influenza. However, our study shows that testing is associated with prescription, suggesting that increased testing could help solve the overall problem of low treatment rates.

Moreover, we found several differences in the proportion of antiviral prescriptions based on patient factors, study sites, and influenza season. Children who were younger and had certain underlying conditions were more likely to be prescribed antivirals, which aligns with clinical guidelines to treat patients at higher risk. However, even within the higher risk group, over 2/3 of children were not prescribed an antiviral. In addition, considerable site variation in antiviral treatment highlights the fact that prescription practices may vary substantially across different ED settings. This is similar to another study that reported wide variation in the use of influenza antivirals among children in the United States [16]. We also saw a temporal trend, with higher odds of prescription in later influenza seasons, such as the 2019–2020 season, as compared to the 2016–2017 season. This is coincident with updated guidance from the CDC and IDSA in 2018 and 2019 and could suggest that guideline adherence improved in response to these new releases [2, 5, 6]. Additionally, the 2017–2018 and 2019–2020 influenza seasons were classified as seasons with high severity in children, which may have led to increased antiviral use [17].

Many studies have identified several possible contributors to low usage of antivirals, including varying perceptions of antiviral effectiveness, hesitation in prescribing antivirals outside the optimal 2‐day window or without a positive influenza test, concern for potential side effects associated with antiviral use such as vomiting and diarrhea, cost of treatment, and misunderstandings of treatment guidelines [7, 8, 16, 18, 19]. These gaps reveal an urgent need for interventions that improve adherence to pediatric antiviral treatment protocols, especially among patients at higher risk of severe influenza. Specific opportunities warranting further research include healthcare provider education and training, enhanced patient access to timely antiviral treatment, clinical decision support tools, and electronic medical record alerts that facilitate appropriate prescribing. Further, family, patient, and community education on the benefits of influenza antiviral treatment among high‐risk children could help improve adherence. Moreover, we found that less than half of our study population received the current years' influenza vaccine. This is especially concerning for children at higher risk of severe influenza illness. Increasing the rate of pediatric influenza vaccine uptake is also crucial for ensuring children have protection against influenza. A coordinated effort across these areas may help address the complex factors underpinning the current under‐use of antivirals and thereby improve outcomes for pediatric patients.

Strengths of this study include robustness of the data, active surveillance design, and systematic molecular testing for all enrolled children. The study is subject to some limitations. First, although we used data from seven sites which represent large, academic‐based US EDs, they may not be representative of the seasonal influenza burden or the clinical prescribing practices of antivirals across all US states, especially since our study indicated large differences in prescribing patterns by site. Additionally, data were collected before the COVID‐19 pandemic, and current testing and prescribing patterns subsequently may have changed. However, another study analyzing antiviral prescribing during the 2022–2023 season identified that pediatric hospitalized patients had less antiviral use than seasons prior to the COVID‐19 pandemic [20]. Third, we did not identify all children who may have been at higher risk for severe influenza, including those who were obese, used long‐term aspirin medication, were pregnant, or were of American Indian or Alaska Native origin, as these conditions were either uncommon in our dataset or the necessary data were unavailable for assessment. Fourth, we only had information on whether an antiviral was prescribed, not if the prescription was filled by a pharmacy, nor could we ascertain whether hesitancy from family members influenced antiviral prescription. In addition, cost of antiviral therapy could not be assessed in this study. Furthermore, treatment outcomes could not be assessed due to the nature of the cross‐sectional study design. Lastly, the NVSN study design was constrained by a couple limitations. First, certain NVSN sites limited ED enrollment to children < 5 years old during portions of the study timeframe. Second, the overall surveillance for the study commenced on December 1, 2016; consequently, the 2016–2017 influenza season was shorter, potentially leading to a decrease in the detection of early influenza cases. Finally, for children who received the current season's influenza vaccine, we cannot determine whether sufficient time elapsed for the vaccine to provide protection.

## 5 Conclusion

Influenza antivirals for children continue to be under‐prescribed in the ED, including for children at higher risk of severe influenza. Increased efforts are warranted to promote appropriate antiviral prescribing for children with influenza. In addition, further research is needed to understand treatment decision‐making in the pediatric ED setting and barriers to antiviral use toward improving adherence to clinical guidelines. Our study highlights the concerning gap in evidence‐based influenza treatment of children in the ED setting, similar to that in hospitalized children with influenza, and underscores the need for implementation research to increase adherence.

## Author Contributions

Tess Stopczynski conceptualized the research question, curated the data, contributed to the investigation, methodology and visualization, wrote the original draft, and critically reviewed and edited the manuscript. Justin Z. Amarin conceptualized the research question, curated the data, conducted formal analysis, developed the methodology, performed visualization, and participated in writing, reviewing, and editing the manuscript. James W. Antoon and Olla Hamdan conceptualized the research question, contributed to data interpretation, and critically reviewed and edited the manuscript. Laura S. Stewart, James Chappell, Eileen J. Klein, Janet A. Englund, Geoffrey A. Weinberg, Peter G. Szilagyi, John V. Williams, Marian G. Michaels, Julie Boom, Leila C. Sahni, Mary Allen Staat, Elizabeth P. Schlaudecker, Jennifer E. Schuster, Rangaraj Selvarangan, Christopher J. Harrison, Heidi L. Moline, Ariana P. Toepfer, and Angela P. Campbell designed data collection instruments, coordinated and supervised project administration, data collection and testing, and reviewed and edited the manuscript for important intellectual content. Andrew J. Spieker conducted formal analysis, developed methodology, and critically reviewed and edited the manuscript. Samantha M. Olson conceptualized and designed the study, designed data collection instruments, coordinated and supervised project administration, data collection and testing, conducted investigation, and critically reviewed and edited the manuscript. Natasha B. Halasa conceptualized and designed the study and research question, conducted investigation, designed data collection instruments, coordinated, and supervised project administration, data collection and testing, contributed to data interpretation, provided supervision, and critically reviewed and edited the manuscript.

## Conflicts of Interest

J.W.A. previously served as a member of the Scientific Advisory Board for AstraZeneca. G.A.W. has received honoraria from Merck & Company for writing and revising chapters in the Merck Manual EPS, and M.A.S. receive research funding from Pfizer and honoraria from Sanofi Pasteur for vaccine consultation. J.A.E. reports work as a consultant to Abbvie, Astra Zeneca, Meissa Vaccines, Pfizer, Moderna, and Sanofi Pasteur and research to support university from AstraZeneca, GlaxoSmithKline, Merck, and Pfizer. M.G.M. reports research to support university from Merk Sharpe & Dohme and non‐financial research grant from Viracor. J.E.S. reports work as a consultant at AAMC. J.V.W. previously served as a member of the Scientific Advisory Board for Quidel and on an Independent Data Monitoring Committee for GlaxoSmithKline. S.M.O. reports travel support from the Gates Foundation. N.B.H. received grant support from Sanofi and Quidel, received current grant support from Merck, and served on an advisory board for CSL Seqirus.

## Peer Review

The peer review history for this article is available at https://www.webofscience.com/api/gateway/wos/peer‐review/10.1111/irv.70124.

## Disclaimer

The findings and conclusions in this report are those of the authors and do not necessarily represent the views of the US Centers for Disease Control and Prevention (CDC).

## Article's Main Point

Among influenza‐positive children who presented to the emergency department, roughly 3/4 were at increased risk for severe influenza illness, of which only a third were prescribed influenza antivirals. Treatment of influenza by national recommendations is suboptimal in this population.

## Supporting information


**TABLE S1.** Adjusted odds ratios of study site for antiviral prescription among children at higher risk of severe influenza illness presenting to the emergency departments of seven children’s hospitals within the New Vaccine Surveillance Network (*N* = 1931).

## Data Availability

The data that support the findings of this study are available from the corresponding author upon reasonable request.
