# Supplementary material for: Antiviral Prescription in Children With Influenza in US Emergency Departments: New Vaccine Surveillance Network (NVSN), 2016–2020
Source: Influenza Other Respir Viruses. 2025 Jun 10;19(6):e70124. doi: 10.1111/irv.70124 (PMC12152273; doi:10.1111/irv.70124)
Supplement: Supplementary file 1 — TABLE S1. Adjusted odds ratios of study site for antiviral prescription among children at higher risk of severe influenza illness presenting to the emergency departments of seven children’s hospitals within the New Vaccine Surveillance Network (N = 1931). [file IRV-19-e70124-s001.docx]

**Supplementary Material**

**Supplemental Table 1.** Adjusted odds ratios of study site for antiviral prescription among children at higher risk of severe influenza illness presenting to the emergency departments of seven children’s hospitals within the New Vaccine Surveillance Network (*N*=1,931).

|  | **Adjusted OR** | **95% CI** | ***p*-value** |
| --- | --- | --- | --- |
| Study site |  |  |  |
| B | Ref. |  |  |
| A | 16.48 | 6.99, 38.31 | **<0.001** |
| C | 11.77 | 4.89, 28.46 | **<0.001** |
| D | 34.62 | 13.73, 87.25 | **<0.001** |
| E | 59.02 | 21.26, 163.84 | **<0.001** |
| F | 14.00 | 5.86, 33.45 | **<0.001** |
| G | 8.04 | 3.15, 20.53 | **<0.001** |
